# A Pre-Adolescent and Adolescent Clinical Sample Study about Suicidal Ideation, Suicide Attempt, and Self-Harming

**DOI:** 10.3390/ejihpe12100100

**Published:** 2022-10-01

**Authors:** Alessia Raffagnato, Sara Iannattone, Rachele Fasolato, Elisa Parolin, Benedetta Ravaglia, Gaia Biscalchin, Annalisa Traverso, Silvia Zanato, Marina Miscioscia, Michela Gatta

**Affiliations:** 1 Department of Woman and Child’s Health, Padua University Hospital, 35128 Padua, Italy; 2 Department of General Psychology, University of Padua, 35131 Padua, Italy; 3 Department of Developmental Psychology and Socialization, University of Padua, 35131 Padua, Italy

**Keywords:** suicidality, inpatients, adolescence, risk factors, nonsuicidal self-injury

## Abstract

Suicide is the second cause of death among adolescents, and nonsuicidal self-injury (NSSI) is one of the main risk factors for suicidal behavior. However, the possible variables specifically associated with suicidal ideation and suicide attempt, as well as the psychopathological characteristics linked to the concomitant presence of suicidal ideation/attempt and NSSI are still under-investigated in youth. The current study aimed to address these issues in a sample of 174 young Italian inpatients (*M*_age_ = 14.3 years ± 1.93, 78.2% girls). Sociodemographic and clinical variables were assessed through psycho-diagnostic interviews and ad hoc questionnaires. A binomial logistic regression was performed to identify the predictors of suicidal ideation and suicide attempt. Then, Kruskal–Wallis tests were run to analyze the psychopathological differences between patients with suicidal ideation and suicide attempt considering the coexistence of NSSI. The results highlighted that previous access to child mental health services and general psychopathological problems significantly predicted suicidal ideation, while previous hospitalizations, borderline personality functioning, and affective disorders significantly predicted suicide attempt. In general, inpatients with also NSSI reported higher levels of internalizing, somatic and total problems, impulsiveness, alexithymia, and emotional dysregulation. The clinical implications of our findings in terms of primary and secondary preventive programs are discussed.

## 1. Introduction

Suicide is the second leading cause of death among people between 10 and 24 years [1,2] and is considered one of the main public health issues [3]. In particular, the literature shows that adolescents generally present suicidal thoughts and attempt suicide more frequently than adults [4], thus highlighting the relevance of this specific developmental stage to investigate and pinpoint risk factors for suicidality. In addition, a study reported that more than a third of adolescents with suicidal ideation have attempted suicide [5], and a Canadian work—based on a sample of approximately 2000 students—revealed that the risk of suicide was 25.5% among those who reported suicidal ideation [6]. However, it often happens that signals of psychological suffering in young people are not recognized; for instance, requests for assistance due to general psycho-physical problems (e.g., psychological interview, psychotherapy, access to a psychiatric unit, eating disorders center, previous hospitalizations, etc.) should not be underestimated because they may be based on a profound malaise that can foster suicidal thoughts and acts [7,8,9,10,11].

The transition from suicidal ideation to suicidal behavior usually occurs within 1–2 years from the onset of suicidal ideation [12]. Over the years, several theories within the ideation-to-action framework have been developed; these theories try to explain the transition from suicidal ideation to suicide attempt by differentiating the variables involved in mere suicidal ideation from those underlying suicide attempt. In particular, Joiner’s theory [13] highlighted the importance of the variable “capability for suicide” as a risk factor for suicide attempts among people with suicidal ideation [14,15].

Generally speaking, different studies have pinpointed the variables that can be associated with suicidality in developmental age, showing that most of them are common to both suicidal ideation and attempt (e.g., [16]).

Regarding the family context, the literature reports that young people with suicidal behavior often have relatives with current or previous psychiatric problems [17,18]. Moreover, the relationship between parents and their children plays a crucial role in influencing the social, emotional, and psychological development of children [19]. A dysfunctional family environment, characterized by low support and high conflict, has been associated with an increasing prevalence of suicidal ideation and suicidal attempt in developmental age [20,21]; as a consequence, the evaluation of the child in his family environment is fundamental [22,23], especially in cases of preexisting psychological difficulties.

About the extra-family environment, the school context should not be underestimated. In fact, school can be an adverse place for youth if bullying occurs, and this is another risk factor for suicidal behavior [24,25,26]; in particular, the association of suicide attempt with being bullied seems greater than with suicidal ideation [27]. Moreover, difficulties in peer relationships are also considered possible “precipitating factors” for suicide, particularly in young people [28]; in fact, being accepted by peers is a fundamental value in adolescence, hence victimization by peers is often perceived as a devastating experience that could lead to depression and suicidal ideation [3].

In addition, traumatic experiences during childhood and a history of childhood abuse and maltreatment were found to contribute to the onset of suicidality in adolescence, both directly and indirectly, through the role of mediators such as post-traumatic stress disorder, depression, emotion dysregulation, low self-esteem, and dissociative symptoms. The diathesis–stress model asserts that stressful life events interact with vulnerability factors and increase the probability of suicidal behavior [27,28]. Moreover, other situations perceived as particularly stressful by the child, such as change of residence, legal issues or sentimental relationship breakdown, can precede suicidal behavior, probably through the development of adaptive problems [29]. In particular, it emerged that adolescents with a history of suicidal behavior have generally been more exposed to stressful life events than those with only ideation [28,30].

Among psychological factors, a meta-analysis found an association between alexithymia and suicidality; specifically, the association between alexithymia (particularly in forms of difficulty in identifying and describing one’s own emotional states) and suicidal ideation resulted in being stronger than that between alexithymia and suicidal attempt; however, the impact of depression on this correlation remained unclear [31].

Another important risk factor for suicidal behavior, particularly in young people under 14 years of age, was found to be impulsiveness [32,33]; more specifically, in a meta-analysis by Liu and colleagues [34], cognitive impulsiveness and motor impulsiveness were shown to have, respectively, a moderate-to-important and mild-to-moderate effect on suicide attempts. However, higher scores on motor impulsiveness emerged when impulsiveness was assessed shortly after the suicide attempt; this result thereby highlights that the assessment of different forms of impulsiveness could enable prompt recognition of subjects more at risk of acting out. However, the literature seems controversial with regard to the association between impulsiveness and suicidality. In a meta-analysis [35], impulsiveness levels were found to be similar between subjects who attempted suicide and those with ideation only, and, at the same time, those who presented more impulsive attempts did not score significantly higher on specific tests [36]. Millner et al. also found similar values of impulsiveness between the two groups, although those who attempted suicide showed higher values of impulsiveness when in a negative situation [37]. 

Pertaining to psychiatric disorders, the relationship between Borderline Personality Disorder (BPD) and suicidal behavior is also relevant: this disorder is often associated with experiences of negative emotions, perceived as intolerable, and impulsive attempts to regulate them [38]. Yen and colleagues conducted a longitudinal study in patients with BPD and showed that approximately a fifth of the patients exhibited a suicidal act during the two-year follow-up [39].

Other psychopathologies to be considered when studying suicidality in developmental age are affective disorders. In particular, epidemiological studies and a meta-analysis confirmed the role of depression as a strong risk factor for suicidal thoughts and for the transition from suicidal ideation to suicide attempt [5,40]. According to Cash and Bridge, in clinical samples of adolescents, approximately 85% of patients with major depressive disorder or dysthymia are at a high risk of presenting suicidal ideation: 32% will attempt suicide during adolescence or early adulthood and 20% will make multiple attempts [41]. Bipolar disorders may be associated with an increased risk of suicidal behavior, too. A review on suicidality in children and adolescents with bipolar disorder found that suicidal ideation and suicide attempts have incidence rates of 25% and 15%, respectively, with a prevalence of 50% for suicidal ideation, and 25% for suicide attempts [42].

Finally, when investigating suicidal ideation and behavior, nonsuicidal self-injury (NSSI)—which is characterized by self-injurious acts without suicidal intent—should also be considered. The prevalence of NSSI in adolescents varies between 7.5% and 46.5% [43,44,45,46] and has increased during the COVID-19 pandemic [47,48,49]; its onset is generally between 12 and 14 years of age [50], but cases of younger people have been reported [51]. Several theories have been proposed on the relationship between different self-injurious phenomena. For example, NSSI and suicide have been considered as two expressions of the same spectrum (i.e., self-harming), with the main difference that self-injurious behaviors in NSSI are not motivated by suicidal intention (e.g., [14]). Moreover, some authors consider NSSI as a risk factor for suicidal ideation and suicide attempt, while others consider it as a protective factor [52,53,54]; in particular, some authors posited that NSSI could be a direct risk factor for suicide, or its influence on suicide could be mediated by other factors, such as depression, suicidal ideation, personality disorders, low self-esteem, or low family support, which can facilitate acting out [55,56,57]. Nevertheless, the role of NSSI in the transition from suicidal ideation to suicide attempt remains controversial.

NSSI, suicidal ideation, and suicide attempt share several risk factors, psychiatric comorbidity, and high prevalence in young people. At a symptomatological and psychopathological level, the main risk factors associated with suicidal and nonsuicidal self-harm are impulsiveness [32,33,58,59], alexithymia [60,61,62,63,64]—considered a transdiagnostic risk factor [65,66,67,68]—somatic problems [69,70,71,72,73,74,75], emotion dysregulation [76,77,78,79,80], affective disorders [81,82,83,84,85], borderline functioning and borderline personality disorder [86,87,88,89]. However, it seems still unclear what differences exist in the psycho-behavioral profiles of patients with both suicidal ideation/attempt and NSSI.

To date, a certain body of evidence has been accumulated on the characteristics of suicidal ideation and suicide attempt in different populations; however, the literature on this topic has some limitations. First, most studies have considered nonclinical samples and examined risk factors for suicidal ideation in general, without differentiating suicidal attempters from ideators [15,90]. Nevertheless, according to the ideation-to-action framework, it is necessary to expand the research on the specific factors associated with suicide attempt or mere suicidal ideation, as several different variables could be involved in the path from suicidal thoughts to suicidal acts [15,91].

Furthermore, most works have focused on the adult population, but young people should be carefully kept in mind since nowadays more and more pre-adolescents and adolescents have access to inpatient and outpatient services for suicidal behavior and thoughts; this notwithstanding, only 20% of the literature has investigated risk factors for suicide in such a population [92]. In particular, to the best of our knowledge, a few studies have examined the risk factors specifically associated with suicidal ideation and suicide attempt in Italian youth [93,94]. The triggers or precipitants of suicidal behavior were found to vary widely across cultures (e.g., [95,96]); hence the importance of exploring the specific risk factors associated with suicidal ideation and suicide attempt in different cultural contexts.

Bearing all this in mind, it seems fundamental to thoroughly investigate the characteristics that could constitute specific risk factors for suicidal ideation or suicide attempt in the pediatric population, in order to better understand and prevent suicide mortality; to obtain the fullest picture possible of this important phenomenon, the differences in terms of psychopathological symptoms according to the concomitant presence of NSSI should also be considered.

### Aims of the Study and Hypotheses

On the basis of the above premises, the present study aimed to further expand the research within the ideation-to-action framework in the Italian context. In particular, our main objectives were:

1. To identify the socio-demographic, clinical, and psychopathological variables specifically associated with suicidal ideation or suicide attempt. To date, the studies investigating this topic have found that most predictors of suicidality seem to be common to both suicidal ideation and attempted suicide (e.g., [16]); however, as stated above, it is important to distinguish the risk factors peculiar to each suicidal phenomenon. In this regard, including some of the variables thought to underlie suicidality in the same model could be particularly useful to establish the statistical significance of each variable over and above the effect of the others. Among the different risk factors common to both suicidal ideation and suicide attempt, we hypothesized that the most relevant for the purpose of our study could be: intra-family problems (e.g., parental conflict, conflictual separation, conflicts among family members; [20]), psychiatric familiarity (e.g., [17]), history of being bullied [27], previous hospitalizations [7], previous requests for assistance or previous access to child mental health services [9], borderline personality functioning (e.g., [93]), general psychopathological difficulties, affective problems (e.g., [92]), impulsivity (e.g., [60]), and alexithyimic traits (e.g., [69]). However, to our knowledge, no previous research has included such variables in the same model yet; therefore, in light of the exploratory nature of this investigation, we did not formulate any definite hypothesis as to which of the above variables could be a specific predictor of suicidal ideation or suicide attempt.

2. To compare patients with suicidal ideation alone and with attempted suicide according to the presence of NSSI. We hypothesized to find greater clinical severity and impairment—expressed in terms of significantly higher scores on all the scales considered—in patients with NSSI, since this could be underpinned by a more severe psychopathology, especially when present in conjunction with other suicidal phenomena.

## 2. Materials and Methods

### 2.1. Participants

Participants were inpatients admitted to a Neuropsychiatry Unit in northern Italy in the period between the 1 January 2015 and the 30 June 2021. We included in the study only inpatients who presented at least suicidal ideation, either as a reason for admission or emerged from clinical history. The final sample resulted composed of 174 individuals aged between 8 and 18 years (*M*_age_ = 14.3 years, *SD* = 1.93). A detailed description of the characteristics of the sample is reported in Section 3.1.

### 2.2. Instruments

The *Barratt’s Impulsiveness Scale-11 (BIS-11)* [97,98,99] measures impulsiveness and includes 30 items divided into three factors (attentional, motor, and nonplanning impulsiveness). By summing these factors, a total score can be obtained: the higher the score, the greater the level of impulsiveness. We used the Italian version adapted for youth by Fossati et al. [100], which showed acceptable psychometric properties (Cronbach’s α = 0.78).

The *Toronto Alexithymia Scale-20 (TAS-20)* [101,102] is a self-report questionnaire that measures the three factors defining alexithymia: Difficulty in Identifying Feelings (DIF), Difficulty in Describing Feelings (DDF), and Externally Oriented Thinking (EOT). Moreover, a total score can also be obtained by summing the scores on the abovementioned factors. The validity and reliability of this measure in the pediatric population have been shown in different studies (e.g., [103,104,105]). Specifically, the Italian version of the TAS-20 has a good reliability, with Cronbach’s α values ranging from 0.52 to 0.75 for the general population, and from 0.54 to 0.82 for clinical samples.

The *Youth Self Report 11–18 (YSR)* [106,107,108] is a self-report tool completed by the adolescent himself. Generally, it is administered to young people of at least 11 years of age; nevertheless, some studies have highlighted its validity and applicability also for children younger than 11 years (e.g., [109,110]). The questionnaire can be split into two different parts: the first section assesses the competences; the second one is composed by 112 items assessing psycho-behavioral tendencies that could represent the manifestation of a psychopathology. From the score on these items, behaviors can be assessed as “normal”, “borderline” or “clinical” on three competence scales (socialization, school, activities) and on eight syndrome scales. Such scales can be grouped into three global scales, which were considered for the purpose of the present study: internalizing problems (anxiety, depression, withdrawal, somatization scales), externalizing problems (aggressive and rule-breaking behavior scales), and other problems (social problems, thought-related problems, attention problems scales). There are also DSM-oriented scales, among which we considered the affective disorders and somatic problems scale only. Finally, we included in the study the Deficient Emotional Self-Regulation (DESR) profile, which can be obtained by summing the scores on the attention problems, anxious/depressed, and aggressive problems scales. Regarding the internal consistency of the tool, Frigerio and colleagues [106] found Cronbach’s α coefficients ranging from 0.83 to 0.91.

### 2.3. Procedures

The present research is a retrospective observational study. It was conducted in accordance with the guidelines of the Declaration of Helsinki and was approved by the local Ethics Committee (protocol code n°0044914/21).

Data for the present study were acquired by reviewing the information contained in patients’ clinical records. Information was collected first during clinical interviews with patients and their parents and then written down in clinical records. Specifically, with regard to the clinical assessment procedure occurring during hospitalization, it is carried out by an interdisciplinary team consisting of medical doctors, psychologists, educators, nurses, and public health social workers. Multidisciplinary evaluation includes medical examinations, diagnostic and therapeutic neuropsychiatric interviews for the patient and clinical interviews for his/her parents, neuropsychological assessment to evaluate the patient’s cognitive and executive functioning, administration of structured and/or semi-structured questionnaires identifying problem behavior and specific symptoms, and the observation of family interactions through the Lausanne Trilogue Play (LTP) procedure [22,111].

The anamnestic Investigation and clinical interviews with the patient and his/her parents consider all the socio-demographic, psychopathological, and clinical-symptomatological factors—also about self-injury (e.g., presence, frequency, and onset of self-injurious acts—described in Table 1 and Table 2, thus enabling clinicians to reconstruct the clinical history of the patient and his/her family, relationship dynamics, and family interactions. The personality organization was established on the basis of the model of personality developed by Kernberg [112]. The author identified three main personality organizations, namely neurotic, borderline, and psychotic. In order to identify the personality organization of the individual, the author proposed the following criteria: identity diffusion degree, capacity to test reality, psychological defense mechanisms and their degree of immaturity. In the present study, personality organization was established using the structural interview based on Kernberg’s criteria [112].

After collecting all the above-mentioned information, through both free and semi-structured interviews, the reference manual for the psychiatric diagnosis of children and adolescents consists of Chapter V (Mental and Behavioural Disorders) of the 10th revision of the International Statistical Classification of Diseases and Related Health Problems (ICD-10; [113]). For clinical and research practice, the Diagnostic and Statistical Manual of Mental Disorders-5 (DSM-5) [114] is also used.

### 2.4. Data Analysis

First, descriptive statistics were calculated to outline the clinical and socio-demographic features of the overall sample, as well as of the specific sub-groups of patients divided according to the presence of suicidal ideation alone (SI) or suicide attempt (SA) (Of note, all patients included in our sample presented with suicidal ideation; therefore, those who attempted suicide also had suicidal ideation. However, for brevity purposes, we named the groups “suicide attempt (SA)” or “suicide attempt + NSSI (SA+NSSI)”, without mentioning suicidal ideation).

Subsequently, a multiple binary logistic regression was conducted to identify the clinical, psychopathological, and socio-demographic variables associated with the presence of SI or SA. The belonging group (SI vs. SA) was the dependent binary variable of the model; then, the choice of predictors was firstly theory-driven: we analyzed the literature on suicidal phenomena in adolescence (see Introduction) and, among the several variables obtained from the patients’ clinical records, we considered only those with a solid theoretical foundation. The subsequent steps were led by general psychometric rules. First, among the variables previously selected, we excluded those with too many levels to limit the number of parameters to estimate and thus improve the model stability (e.g., [115,116,117]). Then, along the same line, we followed the conventional “rule of thumb” of 1:10 ratio between predictors and observations to identify the *maximum* number of predictors to include in the model according to our sample size [115,118,119,120]; such a rule has been considered as a general guideline to both pinpoint an adequate number of predictors and avoid the risk of obtaining an overfitted and unstable model (e.g., [121,122,123]). Subsequently, the final number of variables was chosen in light of the principle of parsimony, so the maximum number of possible predictors was further reduced to simplify the model [118]. This process resulted in the selection of 11 variables, which were entered into three blocks. Block 1 (socio-demographic variables) included: age (as a control variable), intra-family problems (two levels), psychiatric familiarity (two levels), and history of being bullied (two levels). Block 2 (clinical history-related variables) included: previous hospitalizations (two levels), previous requests for assistance or previous access to child mental health services (two levels), and personality functioning (three levels). Finally, Block 3 (psychopathological and symptomatological variables) included: the total problems and affective problems scales of the YSR, and the total scores of the BIS-11 and TAS-20. We checked for the absence of multicollinearity.

Finally, we wanted to investigate the differences between patients who presented suicidal ideation/suicide attempt only and those who also had NSSI; therefore, patients were divided into four groups: suicidal ideation only (SI), suicide attempt (SA)^2^, suicidal ideation and NSSI (SI + NSSI), suicide attempt and NSSI (SA + NSSI)^2^. The Kruskal–Wallis tests were performed to analyze the differences between the above-mentioned groups in terms of psychopathological features, controlling for sex. This test was used since the group sizes were largely heterogeneous (SI: N = 29; SA: N = 24; SI + NSSI: N = 58; SA + NSSI: N = 63), and the literature has shown that such a test enables an excellent control of Type I error rates for both equal and unequal group sizes [124]. The dependent variables were the internalizing problems, externalizing problems, total problems, somatic problems, and DESR scales of the YSR, and all the scales of the TAS-20 and BIS-11. These scales were selected because they evaluate constructs and psychopathological aspects that were found to be related to suicidal phenomena in general, namely impulsiveness and externalizing disorders (e.g., [33]), alexithymia (e.g., [63]), somatic complaints (e.g., [69]), emotion dysregulation (e.g., [77]), and internalizing difficulties (e.g., [83]). For significant effects, Dunn’s post-hoc tests with Bonferroni correction were conducted. Before performing the Kruskal–Wallis tests, we checked the absence of significant differences between the four groups in terms of age, in order to exclude a confounding effect of such a variable (*p* > 0.05).

The 1.6.23 version of the statistical software jamovi [125] was used for data analyses; statistical significance level was established at *p* < 0.05.

## 3. Results

### 3.1. Description of the Characteristics of the Sample

#### 3.1.1. Socio-Demographic, Clinical and Psychopathological Features

The whole sample consisted of 174 hospitalized patients, of which 78.2% were girls. The mean age of girls was 14.4 years (*SD* = 1.72), while that of boys was 14 years (*SD* =1.93).

As regards suicidal phenomena, 29 patients had suicidal ideation alone (17%), 24 also attempted suicide (14%), 58 presented both suicidal ideation and NSSI (33%), and 63 presented both attempted suicide and NSSI (36%). Therefore, 50% of the patients had attempted suicide at least once; the most common methods were ingestion of drug/substances (47%), defenestration or suffocation (45%), and self-cutting (8%).

Moreover, as can be noticed, nonsuicidal self-injurious acts were present in 69.5% of the subjects, of which 50% reported repetitive acts, with a frequency of more than five acts in the previous year. The most common methods were self-cutting, excessive rubbing, hitting, bites, punches, burns, and nearly 60% of patients reported having injured multiple body parts. The reasons for the NSSI acts were self-punishment (11.3%), tension reduction (59.8%), both (8.2%), and other (20.6%), such as agitation, impulsiveness, or the need for attention.

**Figure 1:**
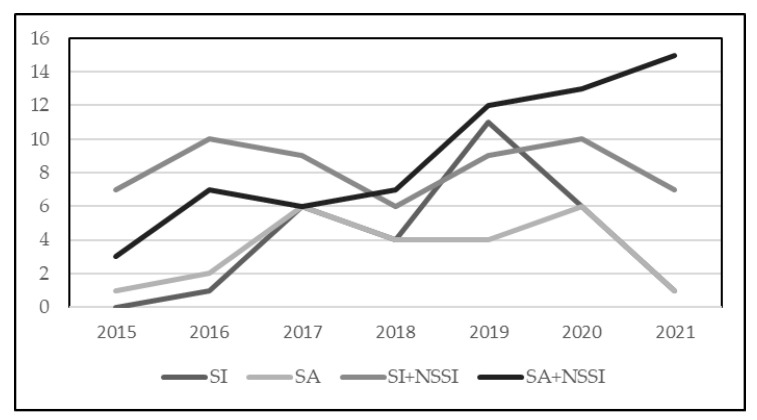
Number of hospitalizations per year according to suicidal ideation (SI), suicide attempt (SA), suicidal ideation and nonsuicidal self-injury (SI + NSSI), and suicide attempt and nonsuicidal self-injury (SA + NSSI).

We analyzed the trend over years of hospitalizations for suicidal phenomena in general: 11 (6.5%) patients were admitted in 2015, 20 (11.5%) in 2016, 27 (15.5%) in 2017, 21 (12.1%) in 2018, 36 (26.7%) in 2019, 35 (20.1%) in 2020, and 24 (13.8%) in the first six months of 2021. Therefore, a general increase in hospitalizations for suicidality was recorded, with some stability between 2019 and 2020; moreover, on the basis of our clinical experience and admission rates for suicidality of previous years, it is possible to hypothesize that the number of hospitalizations in 2021 has roughly doubled by the end of the year, reaching 48. Instead, with regard to specific suicidal phenomena, the number of hospitalizations per year according to the presence of mere ideation, ideation and suicide attempt, and suicidal ideation/suicide attempt together with NSSI is graphically presented in Figure 1: an increase in hospitalizations for suicide attempt with NSSI, stability in hospitalizations for suicidal ideation with NSSI, and a decrease in hospitalizations when NSSI was absent were observed.

In such a picture, the COVID-19 pandemic should be carefully taken into account. Generally speaking, the majority of our sample was composed of patients hospitalized before the pandemic (i.e., 2015–2019; *N* = 115), while a small proportion of participants were hospitalized after the outbreak of the pandemic (i.e., 2020–2021; *N* = 59); specifically, considering 2020 only, patients admitted before the lockdown (i.e., January and February) were 7 (20%), those admitted during the lockdown (i.e., from March to May) were 9 (25.7%), those admitted between June and September were 12 (34.3%), and those admitted between October and December were 7 (20%). Therefore, considering the whole sample, the percentage of patients admitted in the period of implementation of the strictest containment measures was 5.2%. Among the patients hospitalized before the pandemic, the mean age was 14.5 years (*SD* = 1.96); moreover, 19.1% had suicidal ideation only, 14.8% also attempted suicide, 37.7% presented suicidal ideation and NSSI, and 30.4% attempted suicide and reported NSSI. Among those hospitalized after the pandemic, the mean age was instead 14.1 years (*SD* = 1.85); furthermore, 11.9% had suicidal ideation only, 11.9% attempted suicide, 28.8% presented suicidal ideation and NSSI, and 47.5% attempted suicide and also reported NSSI. Therefore, before the pandemic, most patients hospitalized for suicidality reported suicidal ideation and NSSI, while after the pandemic most of the inpatients reported attempted suicide together with NSSI.

The other socio-demographic features of both the whole sample and the two subsamples of patients (i.e., SI and SA) are reported in Table 1. Clinical and psychopathological characteristics are shown in Table 2, while the mean raw scores on the tests administered are shown in Table 3.

#### 3.1.2. ICD-10 Diagnoses and Comorbidities

Diagnoses were established using the ICD-10 [113]. Except for early discharge cases, all patients received at least a diagnosis. Specifically, 59 patients (34.1%) received only a diagnosis, while 72 (46.1%) were diagnosed with a comorbid disorder, 34 (19.7%) had two comorbidities, and 8 (4.6%) had at least three comorbid diagnoses. The main comorbid disorders were neurotic, stress-related, and somatoform disorders (F40-48) (47.9%), mood disorders (F30-29) (17.4%), and behavioral and emotional disorders (F50-59) (14%). When examining the sample divided into SI and SA, 33.3% of SI and 34.4% of SA had only one diagnosis. Furthermore, while 47% of SI patients had only one comorbidity and 19.7% had more than one comorbid disorder, the SA group showed more frequently a higher number of comorbid disorders (≥2 in 26.4% of cases).

When observing the distribution of ICD-10 primary diagnoses in the total sample, the majority (52.6%) were mood disorders, followed by neurotic, stress-related, and somatoform disorders (23.1%), and behavioral and emotional disorders (12.7%), while the other diagnoses were marginally present. The same distribution was observed in the two subgroups (SI vs. SA). In fact, in both groups, most patients presented mood disorders (42.5% in SI, 62.1% in SA), followed by neurotic, stress-related and somatoform disorders (27.6% in SI and 18.4% in SA), and behavioral and emotional disorders with onset usually occurring in childhood and adolescence (16.1% in SI and 9.1% in SA), while the other diagnoses were marginally present.

#### 3.1.3. Psychopharmacology and Post-Discharge Therapeutic Indications

At discharge, 20 patients (11.5%) did not receive any indication to initiate or continue a pharmacological therapy, while 88.5% of patients were prescribed medications: neuroleptic monotherapy in 23% of cases, SSRI monotherapy (selective serotonin reuptake inhibitors) in 5.2% of cases, mood stabilizers in 8%, SSRI + antipsychotics in 24.1%, benzodiazepines (BZD) monotherapy in 1.1%, mood stabilizers in combination with neuroleptic, SSRI and BZD in 5.2%, mood stabilizers in combination with an antipsychotic in 9.8%, SSRI and mood stabilizers 1.1%, BZD plus antipsychotic and mood stabilizer medications in 10.9%.

When the SI group was compared to the SA group, suicide attempters emerged to be prescribed a pharmacological therapy more frequently, mainly with neuroleptics (24.1% of the group) and mood stabilizers (11.4%), in either monotherapy or in combination; patients within the suicide ideation group, on the other hand, were prescribed SSRI in monotherapy (0.1%) or in combination more frequently.

Furthermore, 79.7% of patients were advised to continue their psychotherapy sessions after discharge, both in the SI and in the SA group; moreover, 154 patients (70.9%) received the indication of psychotherapy in combination with pharmacological therapy.

### 3.2. Predictors of Suicidal Ideation and Suicide Attempt

A multiple binary logistic regression model was used to find out the socio-demographic, clinical, and psychopathological features that can represent specific risk factors for suicidal ideation and suicide attempt in young inpatients. The belonging group (i.e., SI vs. SA) was the outcome variable of the model; the reference level was the group with attempted suicide (SA).

Table 4 shows each block of the regression model in detail.

In Block 1 (socio-demographic factors), no significant predictors emerged; the logistic pseudo-R squares (*R*^2^) of the model were Cox and Snell *R*^2^ = 0.04 and Nagelkerke *R*^2^ = 0.05.

When the second block with clinical variables was added, previous requests for assistance or previous access to child mental health services (β = −2.57, *p* = 0.01, odds ratio (OR) = 0.10, CI_OR_ = 0.01–0.64) and previous hospitalizations (β = 1.65, *p* = 0.017, OR = 5.21, CI_OR_ = 1.34–20.2) turned out to be significant. The model *R*^2^ improved to Cox and Snell *R*^2^ = 0.21 and Nagelkerke *R*^2^ = 0.29, thus showing a higher proportion of explained variance accounted for by the model (model comparison: χ^2^ (4) = 13.4, *p* = 0.009).

Finally, psychopathological variables were added in Block 3. The significant predictors emerged to be borderline vs. neurotic personality functioning (β = 2.24, *p* = 0.046, OR = 9.43, CI_OR_ = 1.03–85.9), and the YSR total problems (β = −0.16, *p* = 0.043, OR = 0.85, CI_OR_ = 0.73–0.99) and affective disorders (β = 0.19, *p* = 0.006, OR = 1.21, CI_OR_ = 1.06–1.39) scales; the other significant predictors remained the same as above. The final *R*^2^ for the model increased significantly, with Cox and Snell *R*^2^ = 0.38 and Nagelkerke *R*^2^ = 0.51 (model comparison: χ^2^ (4) = 15.1, *p* = 0.004). Moreover, the Area Under the Curve (AUC) of the final model was 0.86, thus showing a good discriminative ability.

In conclusion, the final model highlighted that previous requests for assistance/previous access to child mental health services and the presence of overall psychological problems were significantly associated with a suicidal ideation outcome, while previous hospitalizations, borderline personality functioning, and the presence of affective disorders were significantly associated with a suicide attempt outcome.

### 3.3. Psychopathological Differences between Patients According to the Presence of NSSI

To study the psychopathological differences between inpatients who presented only suicidal ideation/suicide attempt and those who also presented NSSI, Kruskal–Wallis tests were performed. The independent variables were the belonging group (that is, SI/SA/SI + NSSI/SA + NSSI) and sex (inserted as the control variable), while the dependent variables were the scores on the internalizing problems, externalizing problems, total problems, somatic problems, and DESR scales of the YSR, and the TAS-20 and BIS-11 total and scale scores. Table 5 shows the results of the Kruskal–Wallis tests. As can be seen, significant differences between the groups emerged on all the scales, except for the scale for externalizing problems of the YSR, the scales for motor impulsiveness of the BIS-11, and the EOT scale of the TAS-20. The interaction between sex and group was not significant for any scale, thus showing that the sex variable did not affect significantly the differences between the groups. Significant results of post-hoc tests with Bonferroni correction are displayed in Table 6; since the Kruskal–Wallis test is a rank-based nonparametric tests, we reported both the mean rank and mean raw score of each group. Significant differences emerged between the suicidal ideation group (SI) and the two groups with NSSI (i.e., SI + NSSI and SA + NSSI) as regards the YSR internalizing problems, total problems, and DESR scales, and all the TAS-20 scales; specifically, higher scores on all the scales were obtained by patients who presented NSSI in conjunction with suicidal ideation or suicide attempt (Table 6). With regard to the YSR somatic problems scale, a significant difference was found between the SI group and the SA + NSSI group, with higher scores obtained by the latter (Table 6). Finally, as concerns the nonplanning and attentional impulsiveness scales, and the total score of the BIS-11, significant differences emerged between the SA group and the SA + NSSI group, with higher scores obtained by the latter.

Generally speaking, the presence of NSSI seems to worsen the overall clinical picture of young patients, consistently with our initial hypothesis; in fact, those who presented NSSI together with suicidal ideation or suicide attempt scored significantly higher on the considered scales compared to patients with suicidal ideation or suicide attempt alone.

## 4. Discussion

The main aim of the present work was to investigate the socio-demographic, clinical-symptomatological, and psychopathological features associated with suicidal phenomena in a group of Italian pre-adolescent and adolescent inpatients. Specifically, this research sought to identify the specific risk factors for suicidal ideation or suicide attempt, and to investigate the differences in terms of psychopathological symptoms between patients with only suicide attempt/ideation and those who also presented with NSSI. Indeed, given that suicidality in adolescence is considered a public health problem [3], it seems paramount to pinpoint the possible variables associated with this phenomenon, in order to promptly intervene and prevent suicide mortality in such a vulnerable population.

As regards the description of our sample, a general increase in admissions for suicidality in association with NSSI was observed, in line with the literature that has highlighted a progressive raising in suicidal and nonsuicidal self-harm in young people [2,47]. In particular, some authors have suggested that the exponential increase in NSSI and emergency admissions for suicidal ideation and behavior among young people in the last year may be related to the psychosocial impact of the COVID-19 pandemic [47,48,49]. Our sample was mainly composed of pediatric inpatients hospitalized before the outbreak of the pandemic (i.e., 2015–2019), and the rate of inpatients hospitalized in 2019 is almost equal to that of 2020; therefore, we did not observe an increase in admissions for suicidality from the year immediately before the pandemic to the year of the outbreak of the pandemic. This finding is probably due to the imposed restriction on territorial mental health services and scheduled hospitalizations during the COVID-19 pandemic [49], but it may also be related to the fact that for some young people the effects of the pandemic on mental health have not been immediately observed; in fact, previous studies on children and adolescents with preexisting neuropsychiatric disorders showed a general good adjustment of young patients to the pandemic situation [126,127,128]. However, the long-term negative impact of the pandemic on the psychological well-being of youth could emerge later [129], so we would expect an increase in suicidality rate in the following years. A noteworthy aspect is that most of the patients hospitalized in 2015–2019 reported suicidal ideation and NSSI, while the majority of those hospitalized in 2020–2021 presented suicide attempts and NSSI, thus showing a more severe clinical picture. Future studies in this direction are warranted to better clarify the long-term consequences of the pandemic in terms of suicidality among young people.

Subsequently, we wanted to investigate in more detail the characteristics that can constitute risk factors for suicidal ideation or suicide attempt. The final model showed that the socio-demographic variables considered (i.e., age, intra-family problems, psychiatric familiarity, and being victim of bullying) were not significant predictors of suicidality in general, although it is possible to assume that they may influence the phenomenon indirectly by affecting the environment where pre-adolescents and adolescents live. Among the variables related to the patient’s clinical history and psychopathological condition, previous access to child mental health services and the presence of psycho-behavioral problems were significant predictors of suicidal ideation. Therefore, it may be useful, for preventive purposes, to pay attention to young people who seek some form of assistance (e.g., psychological interview, psychotherapy, access to a psychiatric unit, eating disorders center, etc.) for general psycho-physical, relational, and/or behavioral problems since such a suffering condition could foster suicidal thoughts [7]. This result is in line with the literature highlighting that people who attempt suicide tend to seek help by confiding in family/friends or contacting mental health services [9], and the request often occurs close to the act [8]. Specifically, a recent study has noted that, in the year before suicide, 25.3% of young people had contacted a mental health service and in 9.7% of cases the reason had been NSSI [10]. Therefore, improving the ability to intercept them, listen to them, and give them a reason to trust could act as a barrier against the transition to suicidal action.

Then, previous hospitalizations, regardless of the cause, affective disorders, and borderline personality functioning (vs. neurotic personality functioning) were found to be predictors of an increased risk of suicide attempt. In our sample, one-third of the individuals have previously been hospitalized at least once (for abdominalgia, infection, fever, road trauma, neuropsychiatric disorders). This result is consistent with most published studies showing that suicidal behavior is associated with a higher number of hospitalizations, mainly due to suicidality or affective disorders: these data may indicate greater severity of the clinical condition, fewer personal and family resources, and probably a lower response to therapy [11]. Moreover, the finding about borderline personality functioning aligns with the literature that supports the association between borderline personality functioning/disorder and suicidal attempt in adolescence [86,87,88,89].

Considering the abovementioned results together, it is reasonable to surmise that more specific risk factors, such as affective disorders [81,84], borderline personality functioning, and severe clinical conditions that resulted in previous hospitalizations, may be characteristic of suicidal behavior, while more general and cross-cutting emotional issues and previous access to outpatient services may be related to suicidal ideation.

Then, to investigate the psychopathological differences between patients with suicidal ideation and suicide attempt according to the presence or absence of NSSI, we divided the sample into four groups: (1) suicidal ideation alone, (2) attempted suicide, (3) suicidal ideation and NSSI and 4) attempted suicide and NSSI.

First, the groups with suicidal ideation + NSSI and suicide attempt + NSSI obtained higher scores on all the TAS-20 scales, the YSR internalizing problems and total problems scales, and the DESR profile compared to inpatients with suicidal ideation only. Generally speaking, these findings seem to show that NSSI significantly deteriorates the overall psychopathological profile compared to the mere presence of suicidal ideation.

To be more specific, alexithymia is characterized by the inability to identify and communicate emotions with appropriate words and was found to be a risk factor for several problems in adolescent age, such as risk behaviors (e.g., [65]), social withdrawal (e.g., [68]) somatization (e.g., [66,67]), and suicidal behavior (e.g., [63]). On the basis of the aforementioned results, we assume that general difficulties in emotion regulation and, in particular, in identifying and communicating feelings may be involved in NSSI, thus differentiating adolescents with mere suicidal ideation from those with suicidal ideation/attempt together with NSSI. In particular, people with emotion dysregulation and alexithyimic traits may be more likely to use maladaptive coping strategies; therefore, as also noted in the literature [79], suicidal and nonsuicidal self-harm could be interpreted as a dysfunctional attempt to cope with emotions in the face of an inability to find more functional strategies. To further support this, recent literature has highlighted that improving the ability to identify and describe emotions in individuals who attempted suicide could improve mood and problem solving strategies and reduce states of hopelessness [63].

With regard to internalizing problems, instead, NSSI could be considered a way to recover from negative emotional or cognitive states [80,93], especially if the person has difficulty regulating emotions [64], as suggested above.

Then, our results showed that the group with suicidal attempt and NSSI reported more somatic problems than the group with only suicidal ideation. This finding is consistent with the literature reporting the association between somatic symptoms and both suicidality [69] and NSSI [71]. In particular, somatoform symptoms should be carefully considered because, if not properly investigated and recognized as psychological distress, they can lead to suicidal behavior [72,73]. Not to be forgotten are serious chronic diseases, such as epilepsy, asthma, or the consequences of head trauma, spinal cord injury, which have been associated with an increased risk of suicide [74]. In addition, both NSSI and somatization use the body to express pain or psychological distress [62,66,67,75]. To date, studies on this topic are still too few; nevertheless, there is evidence of an association between these two phenomena: both somatic symptoms and NSSI are often correlated with depressive and anxiety disorders, and their coexistence could be mediated by comorbidity with internalizing problems and alexithymia, thus becoming risk factors for the development of suicidal and nonsuicidal self-injury [85].

Finally, significant differences between patients with SI alone and patients with SA + NSSI emerged in all the above-mentioned variables, too. Generally speaking, we can hypothesize that patients with both SA (and therefore also SI) and NSSI have a more severe clinical pattern than patients with SI alone, thus assuming that a history of NSSI and other clinical factors may play a role in fostering acting out; however, longitudinal studies are needed to corroborate such a hypothesis and assess how said variables interact in the evolution of this phenomenon.

Pertaining to total, attentional, and nonplanning impulsiveness, significant differences emerged between the group with suicide attempts only and the group with suicide attempts + NSSI. This result seems to indicate that a greater level of impulsiveness characterizes patients with NSSI who also attempted suicide. Lockwood and colleagues [59] highlighted that cognitive impulsiveness was associated with the maintenance of NSSI over time, thus leading to a greater risk of suicide attempts. In this scenario, it is possible to surmise that inpatients with NSSI who attempt suicide exhibit a greater tendency to make sudden decisions, without considering different consequences and options; therefore, this behavior could encourage the transition from self-harming to suicidal action. To clarify the relationship between impulsiveness, NSSI, and subsequent suicide attempt, longitudinal studies on clinical and nonclinical samples should be undertaken.

Finally, we would like to read our overall findings in light of the Joiner’s interpersonal theory of suicidal behavior [57]. He stated that social isolation and depressive symptoms may be relevant factors for the transition from NSSI to suicidal action. The results of the present study indicated that, although affective disorders seem to play a crucial role in predicting suicide attempt, the SI and SA groups did not differ significantly in internalizing problems when inpatients with NSSI were excluded from such two groups; therefore, it is possible that NSSI itself contributes to increasing the severity of internalizing disorders. Furthermore, according to Joiner, repetitive NSSI could facilitate the acquisition of the capability for suicide, since the individual becomes used to pain and no longer considers NSSI as a method of regulating emotions [14,91]. Consistently, our results showed greater difficulty regulating emotions in patients with SI + NSSI and SA + NSSI compared to those with SI alone.

The current study presents some limitations. First, the sample size did not enable a homogeneous comparison between different types of suicidal phenomena; moreover, in light of the relatively small sample size, caution must be applied in interpreting and generalizing the results. Second, due to the retrospective observational nature of the study, some data are missing. In addition, self-report questionnaires may lead to some biases linked to social desirability or non-understanding of questions. Data were collected from only one neuropsychiatric unit in northern Italy, thus limiting the generalizability of our results to the whole Italian adolescent population. Further longitudinal studies are required to corroborate our findings, in order to pinpoint the variables associated with suicidal ideation and suicidal attempt in the long-term and investigate the process underlying the transition from suicidal ideation to suicide attempt. In particular, NSSI should be further analyzed to clarify whether it is a specific or aspecific risk factor for suicidal behaviors, as well as its role and that of the other related psychopathological characteristics in fostering suicide attempt. Moreover, protective factors should also be taken into account to develop primary and secondary preventive interventions. Another limitation is related to the wide age range considered in the present study; future investigations should analyze the differences in terms of suicidality according to the different stages of adolescence (i.e., pre-adolescence, early adolescence, middle adolescence, and late adolescence). Moreover, as previously said, the family environment plays a crucial role in the onset of suicidality in developmental age; therefore, further work should describe and distinguish in more detail different family variables associated with suicidality given that we only considered intra-family problems. In conclusion, further studies should better investigate suicidal phenomena in the male population; in fact, the composition of our sample, which consisted mainly of girls, did not enable us to examine the differences between boys and girls in suicidal behavior. However, given that male adolescents manage to complete suicide more frequently, it is relevant to examine the correlates of suicidality in this population for preventive purposes.

## 5. Conclusions

In line with the literature on the epidemiology of suicidal and nonsuicidal self-injury, the current research highlighted an increase in hospitalizations related to self-injury. Some clinical factors associated with suicidality and self-injury were identified: previous access to child mental health services and general psychological problems resulted in being possible risk factors for suicidal ideation, while affective disorders, borderline personality functioning, and previous hospitalizations were more likely in patients who also attempted suicide. Subsequently, the coexistence of suicidal and nonsuicidal self-injury was associated with a more severe psychopathological picture, especially with regard to internalizing problems, impulsiveness, and emotion dysregulation.

In our opinion, the first added value of our research is that it could help identify possible factors involved in the concrete risk of suicide behavior; in fact, since the suicide rate is increasing in the pediatric population, such factors should be detected timely to prevent suicide mortality [93]. Moreover, as regards the Italian context, this research potentially represents a step forward toward a comprehensive understanding of culture-specific patterns in risk factors for suicidality and a starting point for the development of tailored interventions, particularly considering that, in Italy, there are no specialized centers for treating self-harm in developmental age.

Generally speaking, the results of the present study could have valuable clinical implications, since they could promote the implementation of effective interventions to prevent adolescent suicidality. In particular, concerning primary and secondary prevention programs, our study suggests:

- To pay attention, especially among adolescents with suicidal ideation, to those inpatients who exhibit NSSI; this, excluding per se suicidal intention, should be deeply investigated by examining thoughts of death that could not be verbally expressed.
- To organize, for adolescents who require hospitalization, a highly intensive and multidisciplinary post-discharge care, especially in cases of affective disorders, in order to reduce the likelihood of relapse and expression of severe self-injurious behaviors.
- To plan primary level services for reception, support, and social gathering addressed to young people with psycho-physical distress in order to create supporting relationships which could prevent the onset of suicidal ideation or the transition from suicidal ideation to suicidal behavior.

In the background, an appropriate education of health professionals and family members is fundamental to identify in advance adolescents and preadolescents at suicide risk.

## Figures and Tables

**Table 1:** Sociodemographic characteristics of the whole sample and the two subgroups.

Sociodemographic Variables	Total (N = 174)	SI (N = 87)	SA (N = 87)
	*n* (%)	*n* (%)	*n* (%)
**Sex**			
Female	136 (78.2%)	63 (72.4%)	71 (81.6%)
Male	38 (21.8%)	24 (27.6%)	14 (18.4%)
**Ethnicity**			
White	153 (87.9%)	75 (86.2%)	78 (89.7%)
Not White	21 (12.1%)	12 (13.8%)	9 (10.3%)
**Parents’ marital status**			
Married	108 (62.1%)	50 (57.5%)	58 (66.6%)
Separated	57 (32.8%)	31 (35.6%)	26 (30.0%)
Widower	2 (1.1%)	2 (2.3%)	0 (0%)
Other	7 (4.0%)	4 (4.6%)	3 (3.4%)
**Immigration**			
Yes	29 (16.7%)	17 (19.5%)	12 (13.8%)
No	145 (83.3%)	70 (80.5%)	75 (86.2%)
**Siblings**			
Yes	139 (79.9%)	70 (80.5%)	69 (79.3%)
No	35 (20.1%)	17 (19.5%)	18 (20.7%)
**Socialization with peers**			
Good	73 (42.2%)	40 (46.5%)	33 (37.9%)
Difficult/conflictual	74 (42.8%)	34 (39.5%)	40 (46.0%)
Social withdrawal	26 (15.0%)	12 (14.0%)	14 (16.1%)
**School problems**			
Yes	114 (65.5%)	56 (64.4%)	58 (66.7%)
No	60 (34.5%)	31 (35.6%)	29 (33.3%)
**Intra-family problems**			
Yes	116 (66.7%)	58 (66.7%)	58 (66.7%)
No	58 (33.3%)	29 (33.3%)	29 (33.3%)
**Traumatic life events ^1^**			
Yes	104 (60.1%)	52 (60.5%)	52 (60.0%)
No	69 (39.9%)	34 (39.5%)	35 (40.0%)
**Bullying victim**			
Yes	67 (38.5%)	33 (37.9%)	34 (39.1%)
No	107 (61.5%)	54 (62.1%)	53 (60.9%)

Legend: SI = suicidal ideation group, SA = suicide attempt group. Note: Socio-demographic, psychopathological, and clinical-symptomatological data were collected by neuropsychiatrists during the anamnestic investigation included in the general clinical assessment procedure. ^1^ Traumatic life events include, for example, separation/deaths, psychiatric disorders, changes/relocations.

**Table 2:** Psychopathological and clinical characteristics of the whole sample and the two subgroups.

Psychopathological and Clinical Variables	Total (N = 174)	SI (N = 87)	SA (N = 87)
	*n* (%)	*n* (%)	*n* (%)
**Family health problems**			
Yes	108 (62.1%)	49 (56.3%)	59 (67.8%)
No	66 (37.9%)	38 (43.7%)	28 (32.2%)
**Psychiatric familiarity**			
Yes	111 (64.2%)	47 (54.0%)	64 (73.6%)
No	62 (35.8%)	39 (46.0%)	23 (26.4%)
**Chronic illness**			
Yes	59 (33.9%)	35 (40.2%)	24 (27.6%)
No	115 (66.1%)	52 (59.8%)	63 (72.4%)
**Nonsuicidal self-injury**			
Yes	121 (69.5%)	58 (66.7%)	63 (72.4%)
No	53 (30.5%)	29 (33.3%)	24 (27.6%)
**Eating/body-related problems**			
No	89 (51.7%)	45 (51.7%)	46 (52.9%)
Focused	64 (37.2%)	31 (35.7%)	33 (37.9%)
Eating disorders	19 (11.0%)	11 (12.6%)	8 (9.2%)
**Personality functioning**			
Neurotic	49 (37.4%)	27 (42.2%)	22 (32.8%)
Borderline	62 (47.3%)	26 (40.6%)	36 (53.7%)
Psychotic	20 (15.3%)	11 (17.2%)	9 (13.5%)
**Access mode**			
Emergency Room	118 (68.2%)	59 (67.8%)	59 (67.8%)
Outpatient examination	11 (6.4%)	7 (8.0%)	4 (4.7%)
Scheduled admission	11 (6.4%)	7 (8.0%)	4 (4.7%)
Transfer from another hospital	33 (19.1%)	14 (16.2%)	19 (22.8%)
**Access reason**			
Suicidality	91 (52.6%)	26 (29.9%)	65 (74.8%)
Acute anxiety	7 (4.0%)	7 (8.0%)	0
Eating disorders	11 (6.4%)	8 (9.2%)	3 (3.4%)
Psychomotor agitation	22 (12.7%)	13 (14.9%)	9 (10.3%)
Psychotic symptoms	7 (4.0%)	4 (4.6%)	3 (3.4%)
Functional symptoms	6 (3.6%)	6 (6.9%)	0
Nonsuicidal self-injury	18 (9.8%)	13 (14.9%)	5 (5.7%)
Mood disturbance	3 (1.7%)	2 (2.4%)	1 (1.2%)
Other	9 (5.2%)	8 (9.2%)	1 (1.2%)
**Post-discharge services**			
Take in charge at the neuropsychiatry unit	34 (19.5%)	16 (18.4%)	18 (20.7%)
Semi-residential/diurnal care service	13 (7.5%)	9 (10.3%)	4 (4.6%)
Residential care service	8 (4.6%)	4 (4.6%)	4 (4.6%)
District outpatient service	91 (52.3%)	46 (52.9%)	45 (51.7%)
Eating disorders center	5 (2.9%)	4 (4.6%)	1 (1.2%)
Other	23 (13.2%)	8 (9.2%)	15 (17.2%)

Legend: SI = suicidal ideation group, SA = suicide attempt group, NSSI = nonsuicidal self-injury. *Note*: Socio-demographic, psychopathological, and clinical-symptomatological data were collected by neuropsychiatrists during the anamnestic investigation included in the general clinical assessment procedure.

**Table 3:** Mean (*M*) and Standard Deviation (*SD*) of the raw scores obtained on the administered tool considering the whole sample and the two subgroups.

	Total	SI	SA
	*M* (*SD*)	*M* (*SD*)	*M* (*SD*)
**YSR**			
Internalizing problems	72.8 (10.9)	71.3 (11.4)	74.4 (10.1)
Externalizing problems	58.4 (10.9)	57.4 (11)	59.5 (10.7)
Total problems	67.8 (10.7)	66.5 (10.9)	69.3 (10.4)
Affective disorders	76.2 (12.9)	72.9 (12)	79.8 (13.1)
Somatic problems	64.3 (10.4)	63.3 (11.2)	65.3 (9.3)
DESR	197 (29.2)	194 (27.3)	200 (31)
**TAS-20**			
Difficulty Identifying Feeling	25.1 (6.6)	24.4 (7.1)	25.7 (6.2)
Difficulty Describing Feelings	19.4 (3.8)	19.0 (4.5)	19.8 (3.1)
Externally-Oriented Thinking	23.4 (4.9)	22.8 (4.4)	23.8 (5.3)
Total score	67.6 (11.2)	65.6 (12.9)	69.4 (9.4)
**BIS-11**			
Attentional impulsiveness	19.4 (4.2)	19.0 (4)	19.7 (4.4)
Motor impulsiveness	21.6 (4.7)	20.6 (3.7)	22.3 (5.1)
Nonplanning impulsiveness	27.9 (6.2)	26.8 (4.5)	28.5 (7.1)
Total score	68.6 (12)	66.3 (8.5)	70.1 (13.6)

Legend: YSR = Youth Self Report 11–18, TAS-20 = Toronto Alexithymia Scale-20, BIS-11 = Barratt’s Impulsiveness Scale-11, SI = suicidal ideation group, SA = suicide attempt group, DESR = Deficient Emotional Self-Regulation.

**Table 4:** Regression blocks.

	Block 1			+Block 2			+Block 3		
	β	*SE*	*p*	β	*SE*	*p*	β	*SE*	*p*
(Intercept)	4.35	3.30	0.18	7.39	4.19	0.08	−1.13	5.48	0.83
Age	−0.22	0.21	0.40	−0.37	0.25	0.13	−0.31	0.29	0.27
Intra-family problems	−0.59	0.57	0.30	−0.38	0.66	0.57	−0.14	0.90	0.87
Psychiatric familiarity	−0.49	0.58	0.40	−0.36	0.67	0.59	0.48	0.82	0.55
Victim of bullying	0.01	0.53	0.97	0.22	0.60	0.70	0.97	0.79	0.22
Previous access to child mental health services	-	-	-	−2.56	1.08	0.02	−5.72	2.17	**0.01**
Previous hospitalizations	-	-	-	1.65	0.69	0.02	2.10	0.85	**0.01**
Personality functioning									
Borderline- neurotic	-	-	-	0.80	0.68	0.23	2.24	1.13	**0.04**
Psychotic-neurotic	-	-	-	1.16	1.13	0.30	1.84	1.49	0.21
YSR Total problems	-	-	-	-	-	-	−0.16	0.08	**0.04**
YSR Affective problems	-	-	-	-	-	-	0.19	0.07	**0.01**
TAS−20 Total score	-	-	-	-	-	-	0.04	0.04	0.39
BIS−11 Total score	-	-	-	-	-	-	0.03	0.03	0.34
*R* ^2^_CS_	0.04			0.21			0.38		
*R* ^2^_N_	0.05			0.29			0.51		

Legend: YSR = Youth Self Report 11–18, TAS-20 = Toronto Alexithymia Scale-20, BIS-11 = Barratt’s Impulsiveness Scale-11, *R*^2^_CS_ = Cox and Snell *R*^2^, *R*^2^_N_ = Nagelkerke *R*^2^.

**Table 5:** Results of the Kruskal–Wallis tests.

Scales	H	df	*p*
YSR Internalizing problems	13.3	3	0.004
YSR Externalizing problems	5.53	3	0.137
YSR Total problems	14.1	3	0.003
YSR Somatic problems	8.57	3	0.035
YSR DESR	11.6	3	0.009
BIS-11 Total	12.3	3	0.006
BIS-11 Nonplanning impulsiveness	11.2	3	0.011
BIS-11 Attentional impulsiveness	9.82	3	0.020
BIS-11 Motor impulsiveness	5.85	3	0.119
TAS-20 Total	16.9	3	0.001
TAS-20 DIF	14.3	3	0.003
TAS-20 DDF	7.91	3	0.048
TAS-20 EOT	3.02	3	0.388

Legend: YSR = Youth Self Report 11–18, TAS-20 = Toronto Alexithymia Scale-20, BIS-11 = Barratt’s. Impulsiveness Scale-11, DESR = Deficient Emotional Self-Regulation, DDF = Difficulty Describing. Feelings; DIF = Difficulty Identifying Feelings, EOT = Externally Oriented Thinking.

**Table 6:** Significant results of the post-hoc tests.

Levels	*z*	*p*	Mean Rank	Mean Raw Score (*SE*)
**YSR Internalizing problems**				
SI vs. SI + NSSI	−3.09	0.006	36.9 vs. 68.8	63.3 (2.48) vs. 75.1 (2.22)
SI vs. SA + NSSI	−3.57	0.001	36.9 vs. 73.6	63.3 (2.48) vs. 67.6 (3.67)
**YSR Total problems**				
SI vs. SI + NSSI	−2.96	0.009	36.9 vs. 67.9	58.7 (2.45) vs. 70.2 (2.19)
SI vs. SA + NSSI	−3.57	0.001	36.9 vs. 74.4	58.7 (2.45) vs. 64.5 (3.64)
**YSR Somatic problems**				
SI vs. SA + NSSI	−2.91	0.011	41.6 vs. 72	58.6 (2.48) vs. 61.8 (3.69)
**YSR DESR**				
SI vs. SI + NSSI	−2.99	0.008	41.5 vs. 69.5	176.8 (7.11) vs. 202.8 (6.15)
SI vs. SA + NSSI	−3.21	0.004	21.7 vs. 69.4	176.8 (7.11) vs. 191.8 (10.2)
**BIS-11 Total**				
SA vs. SA + NSSI	−2.81	0.015	24.8 vs. 48.4	60.7 (3.97) vs. 74.2 (2.74)
**BIS-11 Nonplanning impulsiveness**				
SA vs. SA + NSSI	−3.11	0.006	21.5 vs. 48.4	23 (2.09) vs. 29.8 (1.44)
**BIS-11 Attentional impulsiveness**				
SA vs. SA + NSSI	−2.57	0.030	23.8 vs. 45.7	16.8 (1.42) vs. 21.3 (0.99)
**TAS-20 Total**				
SI vs. SI + NSSI	−3.25	0.003	19.4 vs. 50.8	53.1 (3.22) vs. 67 (2.28)
SI vs. SA + NSSI	−3.60	<0.001	19.4 vs. 53.24	53.1 (3.22) vs. 68.4 (2.29)
**TAS-20 DIF**				
SI vs. SI + NSSI	−2.87	0.012	21.3 vs. 49.8	18.9 (2.14) vs. 25.6 (1.42)
SI vs. SA + NSSI	−3.17	0.005	21.3 vs. 52.1	18.9 (2.14) vs. 26.7 (1.44)
**TAS-20 DDF**				
SI vs. SI + NSSI	−2.65	0.024	23.3 vs. 49.6	15.3 (1.29) vs. 19.3 (0.86)
SI vs. SA + NSSI	−2.64	0.025	23.3 vs. 48.8	15.3 (1.29) vs. 19.3 (0.87)

Legend: SI = suicidal ideation, SA = suicide attempt, NSSI = nonsuicidal self-injury, YSR = Youth Self Report 11–18, TAS-20 = Toronto Alexithymia Scale-20, DESR = Deficient Emotional Self-Regulation, DDF = Difficulty Describing Feelings; DIF = Difficulty Identifying Feelings; BIS-11 = Barratt’s Impulsiveness Scale-11.

## Data Availability

Data presented in this study are available on reasonable request to the corresponding author. Data are not publicly available because they report private information about participants.
